# Optimization of the Parameters Influencing the Antioxidant Activity and Concentration of Carotenoids Extracted from Pumpkin Peel Using a Central Composite Design

**DOI:** 10.3390/plants13111447

**Published:** 2024-05-23

**Authors:** Roxana Nicoleta Gavril (Rațu), Oana Emilia Constantin, Elena Enachi, Florina Stoica, Florin Daniel Lipșa, Nicoleta Stănciuc, Iuliana Aprodu, Gabriela Râpeanu

**Affiliations:** 1 Department of Food Technologies, Faculty of Agriculture, “Ion Ionescu de la Brad” University of Life Sciences, 3 Mihail Sadoveanu Alley, 700489 Iasi, Romania; roxana.ratu@iuls.ro (R.N.G.); florin.lipsa@iuls.ro (F.D.L.); 2 Department of Food Science, Food Engineering, Biotechnology and Aquaculture, Faculty of Food Science and Engineering, Dunărea de Jos University of Galati, 800201 Galați, Romania; emilia.constantin@ugal.ro (O.E.C.); elena.enachi@ugal.ro (E.E.); nicoleta.stanciuc@ugal.ro (N.S.); iuliana.aprodu@ugal.ro (I.A.); 3 Department of Pedotechnics, Faculty of Agriculture, “Ion Ionescu de la Brad” University of Life Sciences, 3 Mihail Sadoveanu Alley, 700489 Iasi, Romania; florina.stoica@iuls.ro

**Keywords:** carotenoid extraction, antioxidant activity, pumpkin peel, CCD

## Abstract

It has been discovered that the peel of a pumpkin (*Cucurbita maxima*), regarded as a waste product of pumpkin processing, has significant amounts of carotenoids and other antioxidants. This study aims to identify the most effective extraction parameters for an ultrasonic-assisted extraction method to extract the total carotenoids (TCs) and assess the antioxidant activity (AA) of pumpkin peel. To determine the effects of the extraction time, temperature, and material-to-solvent ratio on the recovery of TCs and AA, a response surface methodology utilizing the central composite design (CCD) was used. The extraction temperature (6.25–98.75 °C), extraction duration (13.98–128.98 min), and solvent ratio (0.23–50.23 mL) were the variables studied in the coded form of the experimental plan. The carotenoid concentration varied from 0.53 to 1.06 mg/g DW, while the AA varied from 0.34 to 7.28 µM TE/g DW. The findings indicated that the optimal extraction parameters were an 80 °C temperature, a 10 mL solvent ratio, and a 100 min extraction time. The study confirmed that the optimum extraction conditions resulted in an experimental TC yield of 0.97 mg/g DW and an AA of 7.25 µM TE/g DW. Overall, it should be emphasized that the extraction process can be enhanced by setting the operating factors to maximize the model responses.

## 1. Introduction

There has been a notable global demand for plant-derived natural pigments. These pigments are rich in antioxidants and can replace synthetic colors, especially in the food, pharmaceutical, and cosmeceutical sectors. The majority of synthetic colorants have been documented for their adverse impacts on human health, namely their ability to directly cause hyperactivity and allergic reactions in children and other individuals who are sensitive [1]. Modern customers who prioritize their health are increasingly seeking plant-derived natural pigments, particularly for use in food. As a result, researchers are investigating using plant waste to extract bioactive natural pigments [2]. 

Seeds, peels, and pomace are among the wastes and byproducts from fruits and vegetables that come from the food processing industry. The extraction of various bioactive compounds, such as polysaccharides, peptides, dietary fibers, etc., and other plant secondary metabolites like pigments, polyphenols, antioxidants, antimicrobials, etc., can be significantly increased using these efficiently [3]. One of the main pigments, carotenoids, is abundant in food industry wastes and can be investigated as a natural colorant in pharmaceutical, cosmetic, and food applications [2].

Pumpkin, belonging to the *Cucurbitaceae* family, is a vegetable plant with around 27 species, including *Cucurbita maxima, Cucurbita pepo,* and *Cucurbita moschata*. Various pumpkin varieties exhibit differences in form, color, and chemical composition due to geographical conditions; however, they belong to the same species. Pumpkins include carotenoids, polyphenolic compounds, minerals, and vitamin C. Carotenoids are antioxidants, natural coloring agents, and a precursor to vitamin A. Carotenoids are mostly found in pulp and peels [4]. Numerous epidemiological studies have revealed a link between a high dietary intake of carotenoids and a decreased risk of developing chronic illnesses like cancer, neurological disorders, cardiovascular diseases (CVDs), and diseases of the eyes [5]. Moreover, carotenoids possess health-enhancing properties, including fortifying the immune system and facilitating optimal functioning of the reproductive system [6].

Pumpkins and squash are cultivated worldwide on around 3 million hectares, resulting in a total production of 27.832 million tonnes [7]. The global production and consumption of pumpkins, in various cooked, baked, and processed forms, are associated with consumers’ growing desire to obtain a diverse range of nutrients and phytochemicals through a balanced and adequate diet. Therefore, the effective utilization of pumpkin leads to the generation of its byproducts. Additionally, it is important to highlight that the peel, seed, and pulp are secondary products that result from the manufacturing and processing of pumpkins. Pumpkin processing typically results in a pulp content of 72–76%, a peel content of 2.6–16%, and a seed content of 3.1–4.4% [8]. The byproduct fractions are underutilized and are typically used to enrich animal feed [9]. However, because of their elevated phytochemicals, these fractions have economic potential. They may be investigated for a number of other uses, including those that have pharmacological and therapeutic benefits for human health, such as those that are anti-inflammatory, carcinopreventive, analgesic, antibacterial, and antiparasitic [10,11,12,13].

Regarding nutritional composition, pumpkin fruits—their pulp, peel, and seeds—contain minerals, lipids, proteins, carbs, and fiber [13,14]. Phenolic chemicals, fatty acids, essential amino acids, vitamins, terpenoids, saponins, sterols, tocopherols, and carotenoids are also abundant in pumpkin fruits [13,15,16,17]. Furthermore, pumpkin peel is a highly beneficial source of minerals, protein, fibers, and several isoforms of vitamin E. The nutritional content of food is linked to these beneficial substances, as demonstrated by Mala et al. [18]. Their study revealed that peels contain high levels of minerals, including phosphorus and iron, as well as dietary fiber. Nevertheless, the fruit waste contains valuable nutrients and solid material that can be effectively utilized in many ways. In the food and cosmetic industries, pumpkin byproducts can be utilized as a useful source of ingredients for product fortification, as biodegradable food packaging [19,20], and as carriers in encapsulation procedures [19,20,21,22,23]. Because carotenoids are strong antioxidants, they are frequently utilized as natural coloring agents in food applications such as butter, salad dressings, frozen desserts, roasted foods like popcorn, and various beverages [24,25]. Therefore, an opportunity to use pumpkin wastes to produce pigments for the food industry exists due to the increased consumer demand for natural colorants with antioxidant potential [10].

Obtaining pigments with nutraceutical qualities from pumpkin is difficult because of its hydrophobic characteristics and its susceptibility to light, temperature, and oxidation [26]. It is still a matter of research to maximize the number of biologically active substances from food industry byproducts by efficiently optimizing extraction procedures. Numerous research papers over the years have emphasized various approaches to removing bioactive components from pumpkin peel using various extraction techniques [27,28]. Saini and Keum [29] emphasize the significance of employing appropriate methodologies to extract carotenoids from complex matrices like vegetables. They suggest that a mixture of polar and non-polar solvents, such as acetone/hexane or acetone/ethanol/hexane, is the most suitable approach, as it enables the simultaneous extraction of both polar and non-polar carotenoids. The response surface methodology is essential in obtaining highly purified preservation compounds from pumpkin byproducts [30]. Using a one-factor-at-a-time approach for optimization is impractical and time-consuming. Furthermore, the lack of interactions between the factors limits the attainment of accurate optimum conditions. The use of the response surface methodology (RSM) allows for the optimization of multiple extraction parameters, including the extraction temperature, extraction time, and solvent concentration. The RSM is a frequently used statistical tool for time, resource, and cost reduction in process optimization. Additionally, it enables the enhancement of the recovery of preservation substances from pumpkin waste. This approach not only reduces the amount of time and resources required but also offers a more effective and organized plan for improving the extraction procedure [31]. 

Contemporary extraction methods for utilizing food waste and byproducts promote using plant bioactive compounds. These techniques are safe for human health, support green consumerism, and are environmentally friendly by reducing energy consumption, thus aiding in the advancement of a green and circular economy. Effective extraction processes must be developed and optimized to enhance the extraction of essential molecules. Factors such as the matrix, solvent, temperature, pH, liquid–solid ratio, and extraction time are commonly taken into account to enhance the extraction process [32,33,34]. The improvement in extraction through ultrasound is due to the transmission of pressure waves and the subsequent creation of cavitation forces. This leads to the explosive collapse of bubbles, generating localized pressure that causes the rupture of plant tissue and enhances the release of intracellular substances into the solvent. The ultrasound instrument is easy to use and significantly more cost-effective than alternative extraction technologies, such as microwave-assisted and supercritical fluid extractions [35]. Prior research has demonstrated that the utilization of ultrasound in carotenoid extraction can improve effectiveness, decrease the quantity of solvent used, and save time compared to traditional extraction procedures. In comparison to conventional extraction methods, the ultrasound-assisted extraction of lycopene from tomato waste has been demonstrated to occur with shorter extraction durations, lower temperatures, and smaller solvent volumes with higher extraction yields [36].

In the present work, the response surface methodology was utilized to extract refined preserving substances from pumpkin peels to improve the extraction process’s effectiveness, scalability, and reproducibility. This study conducted a thorough examination of the optimal extraction conditions for obtaining purified preservation components from pumpkin peels (carotenoids) and also assessed the antioxidant activity of these compounds.

This study examined how an ultrasound-assisted extraction technique and varying parameters such as temperature, duration, and solvent ratio affected the recovery of total carotenoids and assessed the antioxidant capacity of pumpkin peel. A central composite design (CCD) was used to optimize the extraction method and improve the carotenoid content and antioxidant activity of pumpkin peel. In summary, the utilization of the response surface methodology was demonstrated to be a valuable technique for determining the optimal conditions that maximize the extraction efficiency of carotenoids and enhance the antioxidant capacity of the resulting extracts. This study emphasizes the significance of utilizing pumpkin fruit waste to recover bioactive compounds and enhance the value of the crop. It addresses the environmental concerns caused by the improper management of crop byproducts.

## 2. Results and Discussions

### 2.1. HPLC Analysis for Carotenoid Compounds

A chromatographic investigation based on the HPLC technique was conducted to characterize the pumpkin carotenoid profile. 

**Figure 1:**
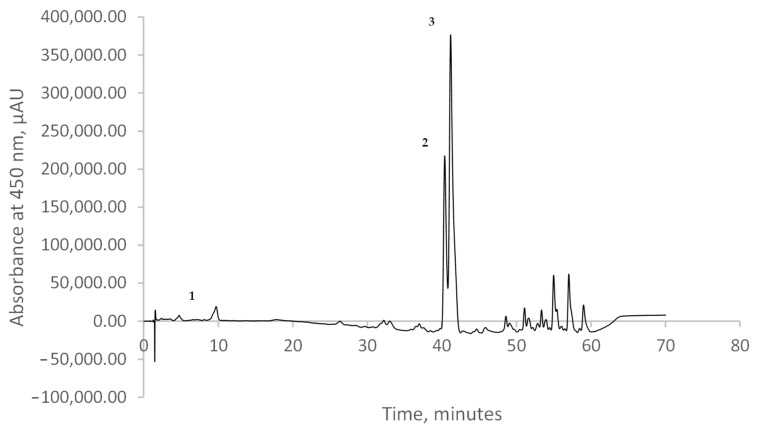
The chromatographic profile of the pumpkin extract at 450 nm: lutein (1), α-carotene (2), and β-carotene (3).

The chromatographic profile of the sample, shown in Figure 1, displayed the presence of several peaks identified at 450 nm. Nonetheless, two major compounds were separated and identified following extraction. Three main compounds were identified: lutein (peak 1), α-carotene (peak 2), and β-carotene (peak 3). The content of lutein was 2.13% (corresponding to a concentration of 43.03 µg/g DW). In contrast, the content of α-carotene was 17.38% and that of β-carotene was 41.82% (corresponding to a concentration of 351.05 µg/g DW and 844.73 µg/g DW) of the total carotenoids in the pumpkin peel extract. Based on their retention time and the data reported in the literature, the other compounds depicted in the chromatogram might presumptively be isomers and derivatives of the main identified carotenoids, especially carotene. In comparison, Provesi et al. [37] studied pumpkin extract and puree and identified β-carotene as the major carotenoid fraction, with a content between 13.38 and 19.45 µg/g sample, whereas α-carotene presented a content between 0.43 and 12.60 µg/g sample. Ninčević Grassino et al. [38] studied two species of pumpkin and revealed that the major carotenoids identified were β-carotene, α-carotene, lutein, and zeaxanthin. The authors also identified numerous ester forms that were measured as traces when reported as compared to the total carotenoid content.

### 2.2. Fitting the Response Surface Models

A central composite design (CCD) and surface response modeling were used to determine the optimal parameters for improving the extraction process. The content of TCs and AA was also determined. The complete CCD matrix used to optimize the principal variables evaluated and the corresponding values are shown in Table 1. 

### 2.3. Influence of the Extraction Parameters on TCs

Carotenoids are a significant class of bioactive substances in pumpkin peels with numerous health-promoting benefits. Thus far, studies have demonstrated a significant variation in the concentration of carotenoids across several *Cucurbitaceae* species and cultivars. Table 1 illustrates how the overall carotenoid content changed depending on different variables, ranging from 0.53 to 1.06 mg/g DW. Regression equations derived from the ANOVA analysis were used to explain the TC values from pumpkin peel, taking into account the variables of the extraction environment (Table 2). The model’s F-value of 705.14 for the TCs from pumpkin peels indicates that this is significant. The results show that model terms are significant if the calculated p-values are smaller than a value of 0.0500. Specifically, A, B, C, AB, AC, A^2^, B^2^, and C^2^ constitute significant model terms. The adjusted R^2^ of 0.9970 and the predicted R^2^ of 0.9888 are reasonably in agreement; the difference is less than 0.2.

R1 (TC) = +0.9137 + 0.0416A + 0.0278B − 0.1443C − 0.0197AB + 0.0572AC + 0.0058BC − 0.0104A^2^ − 0.0084B^2^ − 0.0498C^2^ (1)

Equation (1) represents the model equation that illustrates the relationship between the TCs (R1) and the variables in coded units.

The solvent ratio (C) had the most negative impact on the carotenoid content according to the regression equation’s b coefficients. Furthermore, temperature (A) and extraction time (B) both improved the TCs of the extracts. The extraction of TCs from pumpkin peels was positively influenced, as shown in Equation (1), by interactions between the temperature and solvent ratio (AC) and the time and solvent ratio (BC). Also, moderately negative effects on the TC yield were shown by the interactions between the temperature and extraction time (AB) and the quadratic time of extraction (B^2^), solvent ratio (C^2^), and temperature (A^2^).

**Figure 2:**
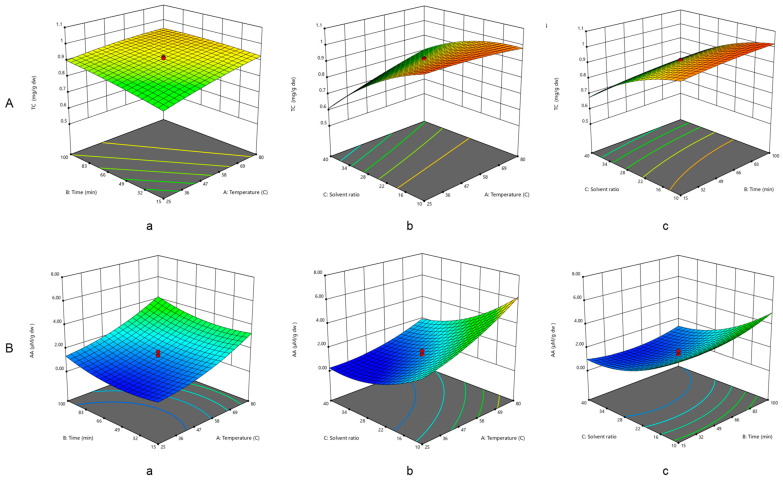
Three-dimensional surface plots screening the variables’ effects on TC (**A**) extraction yield and (**B**) antioxidant activity. ((**A**)—(**a**): temperature–time; (**b**): temperature–solvent ratio; (**c**): time–solvent ratio; (**B**)—(**a**): temperature–time; (**b**): temperature–solvent ratio; (**c**): time–solvent ratio).

A synergistic effect of the independent factors (temperature, time, and solvent ratio) on the TCs of the pumpkin peel extract was discovered through an analysis of Figure 2A(a–c). Second-order contour plots were used to predict the correlation between the independent and dependent variables, as shown in Figure 2A. The three-dimensional response shows how the chosen parameters influence the TCs of the extract.

Extraction temperature and time are the primary factors influencing TC extraction, as Figure 2A(a) demonstrates. The maximum TC value was obtained at 25 °C and around 100 min of extraction time. The maximum carotenoid yield can be obtained at higher temperatures and lower liquid-to-material ratios. Moreover, as Figure 2A(c) illustrates, a lower TC value was produced by shorter extraction durations (57.5 min) and a more excellent solvent ratio (50 mL).

**Figure 3:**
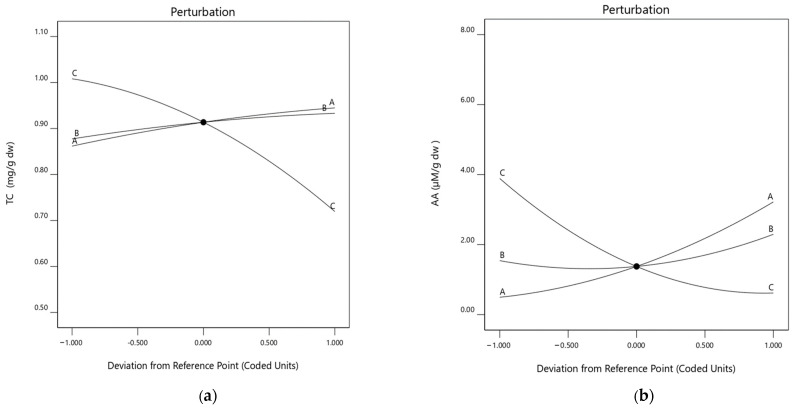
Perturbation graphs representing the effect of each independent variable (A, B, and C) on the pumpkin peel extract’s TCs (**a**) and AA (**b**).

Plots of the perturbations caused by various factors illustrate how they influenced the current response (Figure 3a). To ascertain which elements influence the response most, the perturbation plots contrast the effects of each variable in the design space. A steep slope or curve indicates a factor’s sensitivity to change, whereas a relatively flat line indicates a factor’s insensitivity to change [39]. Curve C on the perturbation graph illustrates the degree to which the solvent ratio value influenced the final result and thus appears to be essential in establishing TCs. Curves A and B, which stood for temperature and time, respectively, demonstrated that solvent ratio had a more significant influence on the extraction than these other factors.

The highest concentration of TCs (1.06 mg/g DW) corresponds to the extracts obtained at 25 °C for 100 min of extraction and 10 mL of solvent ratio (Table 1). Therefore, carotenoid extraction yields may increase over time, and the extracts can easily be introduced into biological systems. 

According to Chuyen et al. [40], the three main variables influencing metabolite extraction in ultrasonic extraction are temperature, extraction time, and the solid/solvent ratio. An increased solid/solvent ratio leads to a shift in the concentration gradient during diffusion, resulting in more efficient plant carotenoid extraction. Carotenoids are expelled from the cell wall, and elevated temperatures harm the cell membrane. Cavitation and mechanical penetration improve the solvent’s ability to penetrate the membrane. Cell rupture and an increase in the mass release of intracellular chemicals into the solvent are caused by acoustic cavitation. The cavitation bubbles accelerate the release of intracellular chemicals that burst at high temperatures, creating a powerful shock wave that permits the solvent to enter the cellular components and break down the cell walls. The swelling and hydration caused by ultrasound cause the cell wall pores to expand [29,41]. Enhancing the dispersion across cellular membranes facilitates extraction [42]. Degradation is the cause of the negative effects of high temperatures on the carotenoid content [35]. These findings are consistent with related investigations on extracting phytochemicals such as carotenoids and other bioactive substances possessing antioxidant properties [43,44,45].

The effectiveness and quality of the extracts in green extraction are greatly influenced by the suitable method, if applicable. In other studies, the experimental carotenoid content was 1.85 mg/100 g dry weight under the optimal conditions of ultrasonic-assisted carotenoid extraction from orange peel using olive oil as a solvent in a 35 min extraction period at 42 °C and a liquid-to-solid ratio of 15 mL/g [46]. The extraction yield was approximately 0.3255 mg carotenoids/100 g of dry peel at the optimal operating conditions (extraction temperature, 51.5 °C; peel/solvent ratio, 0.10; amplitude level, 58.8%; solvent, sunflower oil). This was determined by comparing the effectiveness of ultrasound-assisted and conventional solvent extraction of pomegranate peel carotenoids where vegetable oil types are consumed [35].

Yan et al. [47] investigated the ultrasound-assisted solvent extraction of carotenoids from rapeseed meal using the response surface methodology. The temperature, extraction periods, liquid-to-material ratio, duration, and ultrasonic power were found to impact carotenoid extraction substantially. Under the specified conditions of 49.6 °C temperature, a liquid-to-material ratio of 41.4 mL/g, 48.5 min time, and 253 W ultrasonic power, the average carotenoid yield was 0.1577 ± 0.0014 mg/g, with a 79.61 ± 0.71% extraction level.

Chuyen et al. [48] identified the best conditions for extracting carotenoids and antioxidant capacity from Gac peel utilizing ultrasound-assisted extraction, a Box–Behnken design, and a response surface approach. At the most suitable parameters (76 min extraction time, 50 °C temperature, and 250 W ultrasonic power), the carotenoid yield was 269 mg/100 g DW, and the antioxidant capacity was 822 µM TE/100 g DW. These variations in the carotenoid concentrations of pumpkins can be influenced by various factors such as extraction conditions (solvents and solid-to-solvent ratios), plant species, harvesting season, and site, as well as storage conditions [48,49].

### 2.4. Influence of the Extraction Parameters on AA

The determined antioxidant activity values of the extract from pumpkin peel varied depending on the impact of several variables, ranging from 0.34 to 7.28 mM TE/g DW (Table 1). After the ANOVA explained the antioxidant activity values of the obtained pumpkin peel extract, regression equations were developed based on the variables of the extraction environment (Table 2). The model’s Model F-value of 233.19 for the AA parameter suggests that the model is significant, and p-values less than 0.0500 denote that the model’s terms are significant. The significant model terms in this instance are A, B, C, AC, BC, A^2^, B^2^, and C^2^. 

The regression model employed for DPPH free radical scavenging potential showed a determination coefficient, R^2^, of 0.9953, meaning that the existing model could only specify 0.01 of the variation in antioxidant activity. There was a reasonable agreement between the adjusted determination R^2^ coefficient of 0.9910 and the predicted determination R^2^ coefficient of 0.9734. The model equation for the correlation between the variables in coded units and the antioxidant activity (R2) is shown in Equation (2).

R^2^ (AA) = +1.38+ 1.36A + 0.3760B − 1.64C + 0.0608AB − 0.5104AC − 0.2161BC + 0.4814A^2^ + 0.5412B^2^ + 0.8763C^2^ (2)

The regression equation’s b coefficients revealed that the temperature and extraction time positively impact the antioxidant activity. In contrast, the solvent ratio had a negative effect on the antioxidant activity. Additionally, the interactions between the temperature and solvent ratio (AC) and the time and solvent ratio (BC) significantly negatively affected the antioxidant activity of the pumpkin peel extract. The interaction between the temperature and time (AB) also had a small positive impact. Moreover, the interactions between the quadratic temperature (A^2^), quadratic time (B^2^), and quadratic solvent ratio (C^2^) were all found to have an appreciably positive effect on AA extraction. 

Second-order contour plots (Figure 2B) were utilized to forecast the correlation between the independent and dependent variables and to demonstrate the synergistic effects of the independent factors on the antioxidant activity of the extract. The three-dimensional response area describes the correlative impact of the chosen parameters on the extract’s antioxidant activity. Figure 2B(a–c) display the extraction factors influencing antioxidant activity.

Figure 2Ba shows that temperature and time influence antioxidant activity; AA increases as the volume of the solvent ratio decreases. The maximum antioxidant activity was obtained after nearly 100 min of extraction at a temperature of about 100 °C. Moreover, a decreased solvent ratio and elevated temperature had a beneficial effect on the DPPH free radical scavenging ability, as shown in the graphs (Figure 2Bb). 

Smaller extraction periods and a larger volume of solvent ratio resulted in reduced DPPH free radical scavenging potential (Figure 2Bc).

Furthermore, curve A from the perturbation graph, which shows the notable influence of the temperature of extraction value, was important in determining AA in the perturbation graph. Moreover, curves B and C will have less impact on extraction than curve A (Figure 3b).

This study optimized the ultrasound-assisted extraction process parameters to extract carotenoids from pumpkin peels and enhance their antioxidant activity. While the pro-vitamin A activity associated with certain carotenoids is well known, they also possess potent antioxidant qualities due to their ability to function as conjugated double-bond scavengers of peroxyl radicals and as singlet oxygen quenchers [50]. In vitro spectrophotometric experiments were performed to assess this capability. Three variables (temperature, time, and solvent ratio) were used to optimize the extraction parameters screened through a CCD. Under the optimum conditions, the antioxidant activity was at its highest level at around 80 °C, with a duration extraction of 100 min at a solid-to-solvent ratio of 1 to 10 (g/mL). Higher temperatures and longer extraction times led to an increase in antioxidant activity.

The total carotenoids in pumpkin peel (lycopene and beta-carotene) are known to be heat-sensitive [51]. Significant carotene losses have also been recorded in other materials treated with ultrasound [52,53]. Therefore, the antioxidant capacity may be degraded during the extraction process of pumpkin peel due to high temperatures and high ultrasonic power. 

Corrales et al. [54] obtained comparable results, indicating that ultrasound enhances antioxidant activity compared to traditional extraction techniques. Olive oil was used as a green solvent to enhance the extraction of carotenoids from orange peel using ultrasonic waves. According to the majority of research, applying ultrasonic waves under proper conditions improves the extraction process. Variations in the reaction conditions and the potency of antioxidant components found in carotenoid extracts are the reasons for the variations in the results. The antioxidant activity can be modified by the presence of natural antioxidants (tocopherols) and phenolic chemicals [55].

In their study, Teng and Choi [56] determined that the highest antioxidant capacity of *Rhizoma coptidis* extract extracted with ultrasonic aid was 3.32 mmol/L of TEs at a temperature of 66.22 °C, an extraction period of 46.57 min, and an ethanol content of 59%. 

Hussain et al. [57] measured the DPPH free radical scavenging activity of pumpkin peel, flesh, and seed extracts. The results showed that the pumpkin seed (16.53 mg of ascorbic acid equivalent/100 g) extract had the highest DPPH free radical scavenging activity, followed by the pumpkin peel (13.00 mg of ascorbic acid equivalent/100 g) and flesh (10.58 mg of ascorbic acid equivalent/100 g) extracts.

### 2.5. Optimization and Validation of the Extraction Parameters

**Figure 4:**
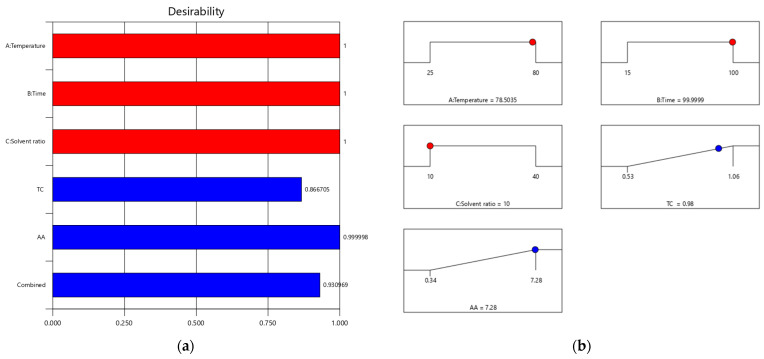
Optimization desirability bar chart (**a**) and ramps (**b**).

The model proposed the optimal factors based on maximizing response desirability to validate the model equation (Figure 4, Table 3). A specific point labeled on the ramp graphs represents the ideal level for the variable under investigation. The desirability function’s value ranges from zero, which is outside the set limits, to one, or a value that is very near to one. The program aims to maximize the function by aiming for the steepest slope feasible, starting at a random point [58]. A desirability score of 0.930 indicated that all the selected conditions were correct. The most effective conditions for achieving the maximum extraction of carotenoids and the strongest antioxidant activity were a solvent ratio of 10 mL, a temperature of 78.5 °C, and an extraction period of 100 min.

The model predicted the maximum concentration of carotenoids and antioxidant activity to be 0.979 mg/g DW and 7.281 µM TE/g DW, respectively. At the same time, the experimental data showed immediate responses to those predicted by the model, particularly 0.97 mg/g DW and 7.25 µM TE/g DW (Table 3). The experimental results showed rapid responses to the model’s predictions. Three extractions were performed under those anticipated variables to validate the model.

## 3. Materials and Methods

### 3.1. Reagents and Chemicals

Acetonitrile, ethyl acetate, n-hexane, acetone, 2,2-diphenyl-1-picrylhydrazyl (DPPH), (±)-6-hydroxy-2,5,7,8-tetramethylchromane-2-carboxylic acid (Trolox), and petroleum ether were obtained from Sigma Aldrich Steinheim (Darmstadt, Germany). All other reagents used in the experiments were of analytical grade.

### 3.2. Pumpkin Peel Preparation

Pumpkin fruit of the species *Cucurbita maxima* cv. *Blue Hubbard* was purchased from a local market in Iasi city. Three pumpkin fruits were cleaned with distilled water, cut into quarters, and peeled using a knife, and their seeds and fibrous material were removed manually. The peels were packed in plastic bags and stored at a temperature of −20 °C. The peels were dried at −42 °C under a pressure of 10 Pa for 48 h in a freeze drier (Alpha 1–4 LD plus equipment CHRIST, Osterode am Harz, Germany) until their moisture level was below 4%, then ground in a lab grinder (powder with 250–500 µm particle sizes). Before being extracted, the powdered samples were vacuum-packed in plastic zipper bags and stored at −20 °C to avoid exposure to light and oxygen.

### 3.3. Ultrasound-Assisted Extraction

Phytochemicals were isolated from pumpkin peel powder utilizing the ultrasound-assisted extraction technique, as reported by Lima et al. [24], with a few minor modifications. To summarize, 10 mL of a 3:1 *v*/*v* n-hexane/acetone solvent mixture was mixed with 1.0 g of pumpkin peel powder. The sample was then ultrasonically treated for 40 min at 40 °C and 40 kHz by Smart MRC LLC, Holon, Israel. The resultant crude extract was recovered and centrifuged for 15 min at 10 °C and 5196 g force. Following separation, the supernatant was gathered, and the residue was repeatedly extracted using 10 mL of n-hexane/acetone (3:1, *v/v*) until it lost its color. Moreover, the Christ AVC 2–18 system (Osterode am Harz, Germany) was used to concentrate the supernatant at 40 °C under reduced pressure. Following the solubilization of the concentrated extracts in the extraction solvent, the total carotenoid (TC) and antioxidant activity (AA) concentrations in pumpkin peel extract were determined. 

### 3.4. Determination of the TC Contents

A spectrophotometric analysis was performed to measure and determine the TC concentrations of the extract, as described by Nistor et al. [59] with slight modifications. In brief, 0.2 mL of the extract was dissolved in the extraction solvent mixture, then introduced into the UV quartz cuvette, and a Libra S22 UV-VIS spectrophotometer was used to measure the absorbance at λ = 450 nm for TCs (Biochrom, Cambridge, UK). The results were reported as mg/g of dry weight (DW). Their concentrations were calculated using the following equation:

(3) $$\mathrm{TCs}\;(\mathrm{mg}/g)=\frac{A\cdot \mathrm{Mw}\cdot \mathrm{Df}}{m\cdot L\cdot \mathrm{Ma}}$$

where A is the sample’s absorbance, Mw is the molecular weight (536.9), Df is the sample’s dilution rate, m is the weight of the concentrated extract, L is the length of the optical path of the cuvette (1 cm), and Ma is the molar absorptivity, which is 2500 L mol^−1^ cm^−1^ for carotenoids. 

### 3.5. Determination of the Antioxidant Activity (AA)

To assess the antioxidant activity of a bioactive substance or extract, it is typically necessary to perform various antioxidant assays. This is because a single compound or a group of compounds may demonstrate varying levels of antioxidative effectiveness across multiple tests [60]. However, the results of our previous investigations on pumpkin peel have demonstrated that carotenoid extracts from pumpkin peel possess DPPH radical scavenging activity, which was found to be significant and also correlated with the total carotenoids in the extract [61]. Therefore, the DPPH radical scavenging activity was chosen to represent the antioxidant activity of carotenoid extracts from pumpkin peel in this study.

The DPPH free radical scavenging method was used to determine the antioxidant activity, expressed as µM Trolox Equivalents (TEs)/g DW [62,63]. Briefly, to measure the in vitro antioxidant activity, 100 µL of the extract was mixed with 3.9 mL of a DPPH stock solution (0.1 M). The mixture was then kept at room temperature for 30 min in complete darkness. The absorbance was determined at 515 nm, and the data were analyzed using a Trolox calibration curve. Then, 100 µL of methanol was used to prepare the blank specimen instead of the sample. The equation for the calibration curve of Trolox was y = 0.45x + 0.0075 and R^2^ = 0.993.

### 3.6. High-Performance Liquid Chromatography (HPLC) Analysis of the Pumpkin Peel Extract

To obtain the chromatographic profile of the obtained extract, a Thermo Finnigan Surveyor HPLC system with a DAD UV–visible detector (Finnigan Surveyor LC, Thermo Scientific, Waltham, MA, USA) was used. The Xcalibur software version 2.0.7 controlled the equipment. The carotenoid compounds from the pumpkin extract were analyzed at 450 nm on a Lichrosorb RP-18 (5 μm) Hibar RT 125–4 column. The elution mobile phase consisted of two solvents, namely 90% acetonitrile (A) and 100% ethyl acetate (B). The injection volume was 20 μL, while the flow rate was 1.000 mL/min. The elution gradient was 0–16 min, 15% B; 16–54 min, 15–62% B; 54–56 min, 62% B; 56–60 min, 62–15% B; 60–70 min, 15% B. The identification and quantification of the main carotenoids were achieved based on the calibration curves of the available standards (lutein, α-carotene, and β-carotene). 

### 3.7. Experimental Design

The central composite design (CCD) approach was chosen to determine the antioxidant activity and experimentally optimize the total carotenoids in the pumpkin peel extract. An experimental factorial model involved a central composite design with five components, three central points, and 20 experimental runs. Table 4 shows the maximum and minimum values of the variables explored in the experimental plan in their current and coded forms. In addition, the CCD creates a quadratic model for response variables.

A second-order polynomial model (5) can be used to represent the software used to test the experimental conditions:

(4) $$R=b_{0}+\sum_{i}^{n}b_{i}\cdot x_{i}+\sum_{i=1}^{n}b_{ii}{\cdot x}_{ii}^{2}+\sum b_{ij}\cdot x_{i}\cdot x_{jd}$$

where R is the predicted response, $b_{0}$ is the intercept, $b_{i}$, $b_{ii}$, and $b_{ij}$ are the regression coefficients, $x_{i}$ and $x_{jd}$ are the independent variables analyzed, and *n* is the number of factors.

### 3.8. Statistical Analysis

In this study, the statistical software Design Expert (v. 12) was utilized to examine the experimental model (Stat-Ease, Inc., Minneapolis, MN, USA). All analyses were carried out in triplicate, and the findings are expressed as mean ± standard deviation. 

## 4. Conclusions

This work utilized a mixture design technique to determine the most effective combination of variables for extracting refined compounds from pumpkin peels, resulting in increased bioactivity. Therefore, a CCD and response surface methodology were used to optimize the ultrasound-assisted extraction process variables (temperature—80 °C; extraction time—100 min; and solvent ratio—10 mL) to obtain pumpkin peel extracts with a high content of TCs and high levels of AA. The interaction of optimal time, temperature, and material-to-solvent ratio improved the extraction of the antioxidant compounds, so our results revealed that the Blue Hubbard peel-refined extract exhibited a notable content of carotenoids (1.06 mg/g DW) and a potent DPPH radical scavenging activity (7.28 µM TE/g DW). 

The results of our study demonstrate the possible positive impacts on the health of the outer layer of pumpkin due to its high concentration of phytochemicals as well as its strong antioxidant properties. These findings emphasize the significance of utilizing this byproduct in the food industry’s circular economy approach. Moreover, our findings display an economically efficient extraction considering the low cost of byproduct materials. Due to the high concentration of functional bioactive components in pumpkin peels, these compounds have a variety of uses in the food, pharmaceutical, and nutraceutical industries. Given that multiple compounds may contribute to the antioxidant activity and bioactive properties of natural matrices, additional research is necessary to enhance the extraction protocols for obtaining specific compounds (such as polyphenols) from pumpkin byproducts. These compounds could potentially serve as innovative colorant agents.

## Figures and Tables

**Table 1:** Matrix of experimental design (real values) with responses in terms of TCs and AA.

Run	Factor 1 A: Temperature (°C)	Factor 2 B: Timp (min)	Factor 3 C: Solvent Ratio (ml)	Response 1 TCs (mg/g DW)	Response 2 AA (µM/g DW)
1	80	15	10	0.97	6.03
2	98.75	57.5	25	0.96	5.12
3	52.5	57.5	25	0.91	1.26
4	80	100	10	0.97	7.28
5	25	15	10	0.96	2.48
6	52.5	57.5	50.23	0.53	0.87
7	52.5	57.5	25	0.92	1.40
8	52.5	57.5	0.27	1.02	6.70
9	25	100	40	0.65	1.01
10	25	100	10	1.06	3.50
11	52.5	128.98	25	0.93	3.57
12	80	100	40	0.82	2.75
13	52.5	57.5	25	0.92	1.24
14	52.5	57.5	25	0.90	1.41
15	52.5	13.98	25	0.87	1.43
16	80	15	40	0.78	2.36
17	52.5	57.5	25	0.91	1.31
18	25	15	40	0.56	0.86
19	52.5	57.5	25	0.92	1.72
20	6.25	57.5	25	0.81	0.34

**Table 2:** ANOVA for the reduced quadratic model for TCs and AA.

TC						AA					
Source	SS	df	MS	F-Value	*p*-Value	Source	SS	df	MS	F-Value	*p*-Value
Model	0.3841	9	0.0427	705.14	<0.0001 ^a^	Model	82.17	9	9.13	233.19	<0.0001 ^a^
A-Temperature	0.0236	1	0.0236	390.02	<0.0001	A-Temperature	25.33	1	25.33	647.00	<0.0001
B-Time	0.0083	1	0.0083	137.29	<0.0001	B-Time	1.52	1	1.52	38.82	<0.0001
C-Solvent ratio	0.2822	1	0.2822	4662.55	<0.0001	C-Solvent ratio	36.26	1	36.26	926.08	<0.0001
AB	0.0031	1	0.0031	51.17	<0.0001	AB	0.0296	1	0.0296	0.7554	0.4052
AC	0.0262	1	0.0262	432.85	<0.0001	AC	2.08	1	2.08	53.22	<0.0001
BC	0.0003	1	0.0003	4.41	0.0621	BC	0.3734	1	0.3734	9.54	0.0115
A²	0.0016	1	0.0016	25.88	0.0005	A²	3.37	1	3.37	86.08	<0.0001
B²	0.0006	1	0.0006	10.68	0.0085	B²	2.67	1	2.67	68.32	<0.0001
C²	0.0351	1	0.0351	579.13	<0.0001	C²	10.84	1	10.84	276.95	<0.0001
Residual	0.0006	10	0.0001			Residual	0.3915	10	0.0392		
Lack of Fit	0.0004	5	0.0001	1.93	0.2445 ^b^	Lack of Fit	0.2383	5	0.0477	1.56	0.3197 ^b^
Pure Error	0.0002	5	0.0000			Pure Error	0.1532	5	0.0306		
Cor Total	0.3847	19				Cor Total	82.56	19			

Sum of squares—SS; mean square—MS; ^a^ significant; ^b^ not significant.

**Table 3:** Validation of the mathematical model.

Dependent Variable	Predicted Value	95% Confidence Intervals	Experimental Value
TCs (mg/g DW)	0.979	0.96–1.01	0.97
AA (µM TE/g DW)	7.281	6.72–7.84	7.25

**Table 4:** Range of values for the factors investigated and encoded values.

Code	Independent Variables	Units	Minimum	Maximum	Coded Low	Coded High
A	Temperature	°C	6.2507	98.7493	−1 = 0.10	+1 = 2.00
B	Time	min	13.9762	128.976	−1 = 20.00	+1 = 60.00
C	Solvent ratio	mL	0.2268	50.2269	−1 = 25.00	+1 = 50.00

## Data Availability

The data supporting this study’s findings are available from the corresponding author (G.R.) upon reasonable request.
